# Seroprevalence and Vaginal Shedding of Herpes Simplex Virus Type 2 in Pregnant Adolescents and Young Women from Morelos, Mexico

**DOI:** 10.3390/v15051122

**Published:** 2023-05-08

**Authors:** Julio Cesar Muñiz-Salgado, Gabriela Juárez-De la Cruz, Dayana Nicté Vergara-Ortega, Santa García-Cisneros, María Olamendi-Portugal, Miguel Ángel Sánchez-Alemán, Antonia Herrera-Ortiz

**Affiliations:** Center for Infectious Diseases Research, National Institute of Public Health, Cuernavaca 62100, Mexico

**Keywords:** HSV-2, vaginal shedding, adolescent pregnancy, seroprevalence

## Abstract

Adolescents and young people are particularly vulnerable to contracting STIs, including HSV-2; furthermore, vaginal shedding of HSV-2 during pregnancy can cause vertical transmission and neonatal herpes. To evaluate the seroprevalence of HSV-2 and vaginal HSV-2 shedding in adolescent and young pregnant women, a cross-sectional study was carried out in 496 pregnant women—adolescents and young women. Venous blood and vaginal exudate samples were taken. The seroprevalence of HSV-2 was determined by ELISA and Western blot. Vaginal HSV-2 shedding was assessed by qPCR of the HSV-2 UL30 gene. The seroprevalence of HSV-2 in the study population was 8.5% (95% CI 6–11), of which 38.1% had vaginal HSV-2 shedding (95% CI 22–53). Young women presented a higher seroprevalence of HSV-2 (12.1%) than adolescents (4.3%), OR = 3.4, 95% CI 1.59–7.23. Frequent alcohol consumption was significantly associated with HSV-2 seroprevalence, OR = 2.9, 95% CI 1.27–6.99. Vaginal HSV-2 shedding is highest in the third trimester of pregnancy, but this difference is not significant. The seroprevalence of HSV-2 in adolescents and young women is similar to that previously reported in other studies. However, the proportion of women with vaginal shedding of HSV-2 is higher during the third trimester of pregnancy, increasing the risk of vertical transmission.

## 1. Introduction

Herpes simplex virus type 2 (HSV-2) is the main causative agent of genital herpes [1]. This infection remains for life and may present periodic reactivations [2]. HSV-2 is widely distributed worldwide, and WHO estimates indicate that HSV-2 affects approximately 13% of people aged 15–49 worldwide (2016 data) [1]. Typically, women are more susceptible than men [3], and pregnant women are more susceptible than non-pregnant women [4,5]. The greatest number of new infections occur during adolescence because adolescents are at high risk of contracting sexually transmitted infections (STIs), due to their behavioral and biological characteristics [6]. Adolescents have risky sexual behaviors, such as multiple sexual partners or lack of condom use [7,8]. Moreover, adolescent women produce less cervical mucus and have low estrogen levels, low vaginal pH, and cervical ectopia [9]. This epithelium is more susceptible to pathogens, unlike the squamous epithelium that lines the vagina and cervix in adult women [9].

HSV-2 infection is difficult to detect because new cases and reactivations are usually asymptomatic [10]. Like all herpes viruses, HSV-2 infection is characterized by cycling through latent and active (or lytic) stages. The active stage is the most important for viral production (shedding) and transmission to new hosts. During pregnancy, HSV-2 reactivation is common, and approximately 5% of pregnant women can transmit the infection, which occurs when the newborn has been exposed to HSV-2 in the genital tract during delivery, causing neonatal herpes [11,12]. Untreated local infection in the newborn can progress to disseminated disease, causing infections of the mouth, eyes, skin, central nervous system, and neonatal abnormalities [11,13].

In Mexico, the seroprevalence of HSV-2 has been reported in women of reproductive age [7,8,14,15,16]. Moreover, the seroprevalence was 14% among pregnant women, higher among pregnant women aged 31 years (26.4%) than among women under 20 years of age (8.6%) [15]. However, data on the percentage of women who vaginally secrete HSV-2 are insufficient, particularly during pregnancy. Therefore, we evaluated the seroprevalence and vaginal shedding of HSV-2 in adolescents and young pregnant women. A high proportion of women in the last trimester of pregnancy were found to have vaginal shedding of HSV-2, which could cause neonatal herpes.

## 2. Materials and Methods

### 2.1. Study Population

A cross-sectional study was carried out in adolescent and young women (10–19 years for adolescents and 20–24 years for young people) who were pregnant and attending health services for pregnancy care in Morelos, Mexico (Yautepec and Cuernavaca Health Centers), during the period from October 2018 to March 2020.

Pregnant women up to 24 years of age were invited to participate, signed the consent/informed assent letter, and answered a questionnaire about sociodemographic, clinical, and sexual behavior characteristics. In addition, they provided vaginal exudate and blood samples. Women who consumed antibiotics for at least two weeks before sampling were excluded from the study.

### 2.2. Ethical Considerations

The protocol was approved by the Ethics, Biosafety, and Research committees of the National Institute of Public Health (Project CI 1529). To obtain informed consent and/or assent, prospective participants were given a written letter explaining the study and detailing the implications of their participation. Likewise, to guarantee confidentiality and anonymity, each participant was identified by a code, omitting her name at all times and without requesting binding data.

### 2.3. Vaginal and Blood Samples Collection

Vaginal exudate was collected with a Dacron swab, which was inserted through the vagina consecutively no deeper than 2 cm, to avoid reaching the cervical region. The swab was placed in a vial with preservation buffer (Sure Path) and stored at −20 °C until processing.

A venous blood sample was also collected and centrifuged at 3500 rpm for 6 min to obtain the serum and prepare aliquots. The samples were stored at −20 °C until processing.

### 2.4. HSV-2 Seroprevalence

Antibodies against HSV-2 were detected with an ELISA (Anti-HSV-2 (IgG2) ELISA (IgG), EUROIMMUN^®^) following the manufacturer’s instructions, using 100 μL of serum diluted 1:101. The plates were washed using a Combiwash Human 18460 plate washer, and absorbance was measured on a Labsystem Multiskan (Model 352, RS-232C series). Samples with indeterminate results by ELISA were processed by a Western blot assay (Anti-HSV-1/HSV-2 IgG-2 EUROLINE-WB (IgG), EURO-IMMUN^®^, Lübeck, Germany following the manufacturer’s instructions.

### 2.5. Vaginal HSV-2 Shedding

Vaginal shedding of HSV-2 was assessed in women who tested seropositive for HSV-2. DNA was extracted from 400 μL of vaginal exudate using the Quick-DNA Miniprep Plus kit (Zymo Research^®^, Irvine, CA, USA). The obtained DNA was quantified in a NanoDrop™ 2000 Spectrophotometer (Thermo Fisher Scientific^®^, Waltham, MA, USA) and processed by qPCR, to amplify a 151 bp fragment of the UL30 gene from HSV-2 and a 140 bp fragment of the human β-globin gene as a control; each gene was amplified separately.

Each 25 µL qPCR contained 1X Maxima SYBR Green/ROX qPCR Master Mix 1, 0.3 mM of each primer, 1 µL of template DNA (≤500 ng), and 10 µL of nuclease-free water. The amplification was carried out in a CFX96TM Real Time PCR Detection System (BIO RAD^®^, Hercules, USA as follows: a cycle of 95 °C for 10 min, 40 cycles of 95 °C for 15 s, annealing temperature (as shown in Table 1) for 30 s and 72 °C for 30 s, and a dissociation cycle of 95 °C at 63 °C, with fluorescence measurements every 2 °C. Each sample was processed in triplicate for both genes, and were considered positive if Ct was >32. The standardization and validation methodology for the relative quantification of the UL30 gene (HSV-2) and the human β-globin gene in anal and genital samples was carried out according to Vergara-Ortega et al. [17].

### 2.6. Statistical Analysis

Definition of variables: The variable “educative level” was divided into three categories—No studies/elementary school, middle school, and high school/university. “Vaginal lesions” refers reported vaginal blisters, ulcers, warts, or pimples at some time in life. “Smoking” refers to having smoked 100 or more cigarettes. “Alcohol consumption” was defined as the consumption of 5 drinks or more weekly. “Drug use” is defined as drugs having been used at least once during their lifetime. Clinical signs or symptoms considered were threatened abortion, dyspareunia, vaginal discharge, and urinary tract infection. The “use of antibiotics” refers to antibiotics taken orally or vaginally prior to the month before the study period. “Hormonal contraceptives” include pills, injections, patches, or morning-after pills. “Frequency of sex” refers to the number of times sexual intercourse was performed in a given period. “Oral sex” referred to oral sex that was received, given, or both.

A descriptive analysis was performed for the demographic, clinical, and sexual behavior variables. Means were determined for the quantitative variables, and frequencies were determined for the qualitative variables, both with 95% confidence intervals. The Shapiro Wilk test was applied to variables to verify the normality of the data. Subsequently, a bivariate analysis was carried out to identify the factors associated with the presence of antibodies against HSV-2. Finally, a multivariate logistic regression analysis was performed from the statistically significant variables (*p* < 0.05), and adjustment was made with the variables “Age”, “Trimester”, “Alcohol consumption”, “Antibiotic use”, “frequency of sex” and frequency of “condom use”, starting from a saturated model until the most parsimonious model was obtained. Statistical analyses were performed using STATA 14.0 software.

## 3. Results

### 3.1. Characteristics of Study Population

The current study included 496 women with a mean age of 19.7 ± 2.9 years, of whom 46.8% were adolescents. Almost half of the pregnant women had a high school education, 26.2% smoked or used to smoke, 43.5% used alcohol occasionally, and 26.2% consumed illicit drugs at some time. A total of 28.0% of the women were in the first trimester of pregnancy, 42.7% in the second trimester, and 29.2% in the third trimester. More than half of the women mentioned some clinical sign related to an STI, 20% reported a history of genital lesions, 10% reported miscarriage or stillbirth, and 26.4% used some form of antibiotic immediately preceding the month before the study period. Almost half of the women considered contracting an STI unlikely. The average number of lifetime sexual partners was 2, 14.9% practiced anal sex, and 38.2% practiced oral sex. A total of 12.9% of women used hormonal contraceptives, and more than half had used a condom at some time in their lives. The demographic, clinical, and sexual behavior characteristics of the pregnant women are shown in Table 2.

### 3.2. Seroprevalence of HSV-2 and Genital Shedding

**Figure 1:**
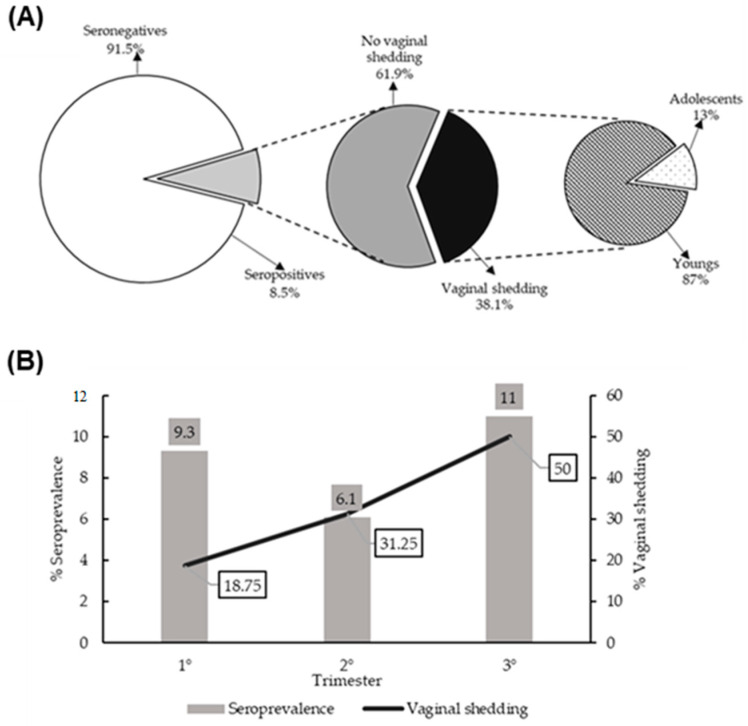
Seroprevalence and vaginal shedding of HSV-2. (**A**) The seroprevalence of HSV-2 in the study population was 8.5%, while vaginal HVS-2 shedding among seropositive women was 38.1%. The vaginal shedding of HSV-2 in young women was 87.5%, and in adolescents was 12.5%. (**B**) HSV-2 seroprevalence stratified by gestational age was 9.3% in the first trimester, 6.1% in the second trimester, and 11% in the third trimester, while vaginal shedding was 18.8% in the first trimester, 31.2% in the second trimester, and 50% in the third trimester.

The seroprevalence of HSV-2 in the study population was 8.5% (95% CI 6–11), while the vaginal shedding rate of HVS-2 among seropositive women was 38.1% (95% CI 22–53), with the majority being young women (87.5%) and not adolescents (12.5%), but this difference was not significant (OR = 3.11, 95% CI 0.57–17.02) (Figure 1A). HSV-2 seroprevalence was found to be higher in the third trimester (11%) than in the first (9.3%) and second trimesters (6.1%), without statistically significant differences (OR = 1.1; 95% CI 0.73–1.70). HSV-2 vaginal shedding was higher in the third trimester (50%) than in the first (18.8%) and second trimester (31.2%), with no statistically significant differences (OR = 1.8; 95% CI 0.82–3.99) (Figure 1B).

### 3.3. Asociated Factors with Seroprevalence of HSV-2

The sociodemographic, clinical, and sexual behavior characteristics of pregnant adolescent and young women, as well as their association with HSV-2 seroprevalence, are shown in Table 3. The seroprevalence of HSV-2 was 3.4 times higher in young women (12.1%) than in adolescents (4.3%). Women with a higher frequency of alcohol consumption had a 2.9 times higher probability of being seropositive for HSV-2. Pregnant women with a higher educative level had the highest HSV-2 seroprevalence, but this difference was not statistically significant. In relation to clinical variables, women who used antibiotics in the last month had a 1.9 times higher probability of being seropositive for HSV-2, showing marginal significance (*p* = 0.07). Women with a history of genital lesions were 1.7 times more likely to have antibodies against HSV-2, but the difference was not statistically significant. Finally, none of the sexual behavior variables were statistically significant.

## 4. Discussion

The seroprevalence of IgG antibodies against HSV-2 found among the 496 pregnant women included in this study was 4.3–8.5% among adolescents and 12.1% among young women. These data are similar to those previously reported. Worldwide, the estimated prevalence is 16.3%. However, among females in the Americas in 2016, it was 7.8% and 14.4% for adolescents and young women, respectively [18]. In Mexico, the HSV-2 seroprevalence ranges from 2.4% to 25%. A study conducted in pregnant and postpartum women from 4 different hospitals in Morelos, Mexico (2006–2009) reported a seroprevalence against HSV-2 of 14.5%, being lower among women 20 years of age or younger than among women 31 years of age or older, with values of 8.6% and 26%, respectively [15]. The national seroprevalence among Mexican men aged 15 to 49 years in 2012 was 9.9%, with a seroprevalence of 2.4% in adolescents and 11.7% in young men, while in women, it was 12.2% [19]. Another study conducted among women seen at public gynecology and colposcopy services, whose mean age was 46 years, reported an HSV-2 seroprevalence of 25% [14]. This report is the first in Mexico focused on HSV-2 in pregnant adolescents and young women, and is the first to include vaginal shedding of HSV-2 during pregnancy.

**Figure 2:**
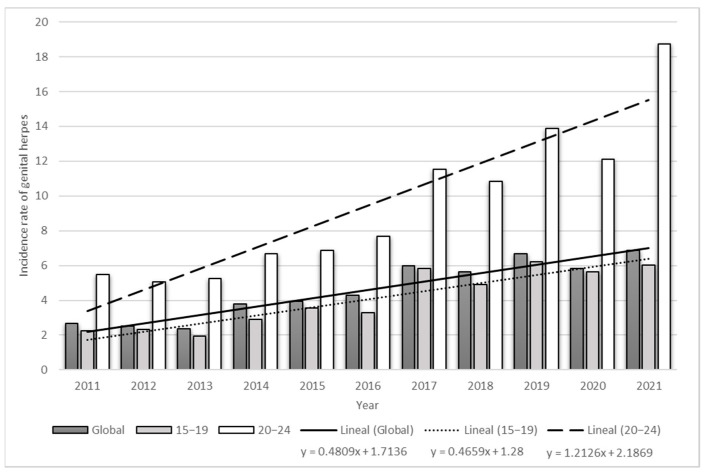
Genital herpes incidence Mexican women, 2011–2021. The bars show the incidence of genital herpes in Mexican women between 2011 and 2021, stratified by age; global is all ages (10 to <65 years). The lines represent the trend line for each stratum. (Elaboration added, 2023, data from reference [20]).

On the other hand, the incidence trend of HSV-2 is increasing in Mexico. Data from the Morbidity Yearbooks of the General Directorate of Epidemiology of the Ministry of Health of Mexico from 2011 to 2021 [20] show that, in 2011, the incidence in women was 2.69 cases/100,000 and was higher among women aged 20 to 24 years (5.47 cases/100,000), while in 2021, the incidence increased to 6.89 and 18.73 cases/100,000, respectively (Figure 2). In the last 10 years, the incidence of HSV-2 has tripled among Mexican women. Women ages 20–24 had an average annual increase of 1.21 cases of genital herpes, more than double the average annual increase for women of 0.48 cases. The slopes are shown in Figure 2. Therefore, young women present a greater possibility of transmitting neonatal herpes during pregnancy.

Risk factors that have been previously associated with HSV-2 infection in women have been age, region, level of urbanization, sexual debut age, educative level, and previous history of abortion [16,20]. However, in this study, only age, alcohol consumption, and antibiotic use were found to be associated with HSV-2 seroprevalence.

In this study, we found that pregnant young women had a higher seroprevalence of HSV-2 than pregnant adolescents, probably due to a longer exposure time to the virus [21]; furthermore, IgG antibodies against HSV-2 indicate past exposure and an immunologic memory of infection [22]. Alcohol consumption was associated with a higher seroprevalence of HSV-2, probably due to the microenvironment of adolescents, which in turn influences their group of possible sexual partners and the probability that these partners are HSV-2 positive [23]. Excessive alcohol consumption is associated with behavioral changes; women who drink before having sexual relations could have risky sexual behaviors, which increases the risk of infection with HSV-2 and other STIs [24]. Interventions to reduce alcohol consumption in adolescents and young women could reduce risky sexual practices and HSV-2 transmission. 

The use of antibiotics was also found to be associated (without adjusting for a higher seroprevalence), probably due to the high prevalence of urinary tract infections in the population, since women are more susceptible to urinary tract infections due to the shorter urethra, the greater proximity of the anus with the vagina, and the easier entry of pathogenic microorganisms through sexual activity and pregnancy per se [25]. Changes in the vaginal microbiota may also be associated with HSV-2 seropositive women, but this issue needs to be investigated.

Regarding educative level, young women with a high school or university education had the highest HSV-2 seroprevalence compared to those in primary or secondary education, with no statistically significant difference; however, a protective association with educative level has been reported: the higher the level of education, the lower the probability that women will acquire STIs such as HIV and HSV-2 [26,27]. Young women who attend school longer have a lower risk of acquiring HIV and HSV-2 infection due to the structure of their sexual network [27], since the number of sexual partners and the age difference between their potential partners are reduced [28]. Although this study found data contrary to what has been reported, the relationship between educative level and HSV-2 seroprevalence in young women may be due more to the age of the women than to their educative level.

Interestingly, although HSV-2 seroprevalence did not significantly differ by gestational age, we found a higher seroprevalence among women in the third trimester of pregnancy. This increase in HSV-2 seroprevalence in the last trimester may be due to a primary infection during the first trimester of pregnancy; the evidence shows that the risk of seroconversion to HSV-2 during pregnancy is 1–5%, but can reach 20% in serodiscordant couples, since, during pregnancy, the main risk factors for primary HSV-2 infection are other STIs, length of relationship, and partner’s history of herpes [29]. However, the study did not track or determine HSV-2 viral detection in seronegative women, which limited detection of the women’s seroconversion rate. 

Furthermore, we observed a higher percentage of women with vaginal shedding of HSV-2 among women in the third trimester of pregnancy, which increases the risk of vertical transmission and the development of neonatal herpes. Neonatal herpes is usually acquired during the intrapartum period by exposure of the fetus to the virus in the maternal genital tract [30]. This transmission can occur even in the absence of lesions, due to subclinical viral shedding [31,32]. Therefore, to prevent the transmission of HSV-2 to the newborn, the timely detection of viral excretion is recommended through laboratory confirmation using HSV-2 antibody detection techniques and molecular detection techniques in vaginal swabs in women with suspected vaginal infection [31,32,33].

Unfortunately, in Mexico, neonatal herpes is not a mandatory reporting disease, so there are no reports on this infection in newborns. Preventing transmission to the newborn by reducing the late-pregnancy acquisition of infection in the mother and altering obstetric management may be the most likely approach to reduce neonatal herpes [30]. However, while the chances of a pregnant woman having an active genital lesion at the time of delivery can be reduced, asymptomatic excretion cannot always be prevented, and the risk of mother-to-child transmission persists [34,35]. Therefore, close monitoring is necessary during pregnancy in adolescents and young women to avoid the development of neonatal herpes.

Among the limitations of this study, ELISA was not used to detect the vaginal dissemination of HSV-2 in women who were seronegative for the virus, and women with primary infection who had not yet generated antibodies may have not been identified. In addition, newborns born to women with vaginal HSV-2 shedding were not followed up for vertical transmission and possible neonatal herpes.

## 5. Conclusions

The seroprevalence of HSV-2 in adolescents and young women in Mexico is similar to that previously reported. However, the proportion of women with vaginal shedding of HSV-2 is higher during the third trimester of pregnancy, increasing the risk of vertical transmission. Close surveillance is needed during adolescent and young pregnancies to prevent the development of neonatal herpes.

## Figures and Tables

**Table 1:** Primer sequences and annealing temperatures for qPCR. Modified from Vergara-Ortega, 2018 [17].

Gen	Primer	Annealing Temperature	Fragment
UL30 (HSV-2)	F1qVHS2 5′-TCA CCG ACA AGG TCA AAC TC-3′ R2qVHS2 5′-ACA CAA TAC TCG CCG ATC AC-3′	63 °C	151 pb
Human β-globina	F2qBETA 5′-GGG CTG TCA TCA CTT AGA CCT CAC-3′ R2qBETA 5′-CCG CTG TCA GAA GCA AAT GTA AGC AAT AG-3’	60 °C	140 pb

**Table 2:** Sociodemographic, clinical, and sexual behavior characteristics and in pregnant adolescents and young women.

Sociodemographic Characteristics		*n*	%
Age	Young	264	53.2
	Adolescents	232	46.8
Educative level	High school/University	181	36.5
	Middle school	247	49.8
	No studies/Elementary	68	13.7
Smoke	Yes/Before	130	26.2
	Never	366	73.8
Alcohol consumption	Frequently	90	18.1
	Occasionally	216	43.5
	Never	190	38.3
Illegal drug use	Ever	130	26.2
	Neither	366	73.8
CLINICAL			
Trimester	3°	145	29.2
	2°	212	42.7
	1°	139	28.0
Clinical signs and symptoms	Yes	287	57.9
	No	209	42.1
Genital lesions	Yes	94	19.0
	No	402	81.0
Abortions/stillbirths	Yes	53	10.7
	No	443	89.3
Antibiotic use	Yes	131	26.4
	No	365	73.6
SEXUAL BEHAVIOR			
STI risk perception	Not probably	252	50.8
	Probably/very probably	244	49.2
Lifetime sexual partners	≥3	157	31.7
	2	121	24.4
	1	218	44.0
Frequency of sex	1–7 times/week	446	89.9
	1–3 times/month	50	10.1
Anal sex	Yes	74	14.9
	No	422	85.1
Oral sex	Yes	190	38.3
	No	306	61.7
Hormonal contraceptives	Yes	64	12.9
	No	432	87.1
Frequency condom use	Never	215	43.3
	Yes, at least sometimes	281	56.7

**Table 3:** Sociodemographic, clinical and sexual behavior characteristics associated to seroprevalence of HSV-2 in pregnant adolescents and young women.

Sociodemographic Characteristics		*n*	% HSV-2	ORc (IC95%)	ORa (IC95%)
Age	Young	264	12.1	**3.1 (1.47–6.38)**	**3.4 (1.59–7.23)**
	Adolescents	232	4.3	1	1
Educative level	High school/University	181	9.4	1.5 (0.49–4.49)	2.0 (0.63–6.45)
	Middle school	247	8.5	1.7 (0.54–5.12)	1.79 (0.58–5.58)
	No studies/Elementary	68	5.9	1	1
Smoke	Yes/Before	130	12.3	1.8 (0.95–3.54)	1.2 (0.55–2.54)
	Never	366	7.1	1	1
Alcohol consumption	Frequently	90	15.6	**2.7 (1.21–6.18)**	**2.9 (1.27–6.99)**
	Occasionally	216	7.4	1.2 (0.55–2.57)	1.2 (0.52–2.57)
	Never	190	6.3	1	1
Illegal drug use	Ever	130	10.0	1.3 (0.65–2.57)	0.9 (0.41–2.02)
	Neither	366	7.9	1	1
CLINICAL					
Trimester	3°	145	11.0	1.2 (0.56–2.60)	1.2 (0.53–2.71)
	2°	212	6.1	0.6 (0.28–1.41)	0.6 (0.27–1.39)
	1°	139	9.4	1	1
Clinical signs and symptoms	Yes	287	8.7	1.1 (0.57–2.05)	0.9 (0.45–1.79)
	No	209	8.1	1	1
Genital lesions	Yes	94	12.8	1.8 (0.89–3.69)	1.7 (0.78–3.59)
	No	402	7.5	1	1
Abortions/stillbirths	Yes	53	7.6	0.9 (0.29–2.54)	0.6 (0.19–1.76)
	No	443	8.6	1	1
Antibiotic use	Yes	131	12.9	**2.0 (1.06–3.89)**	1.9 (0.95–3.79)
	No	365	6.9	1	1
SEXUAL BEHAVIOR					
STI risk perception	Not probably	252	9.9	1.5 (0.77–2.97)	1.3 (0.64–2.56)
	Probably/ very probably	244	6.9	1	1
Lifetime sexual partners	≥3	157	9.6	1.1 (0.54–2.25)	0.7 (0.30–1.54)
	2	121	6.6	0.7 (0.31–1.75)	0.6 (0.30–1.54)
	1	218	8.7	1	
Frequency of sex	1–7 times/week	446	9.2	4.9 (0.67–36.86)	5.84 (0.76–44.7)
	1–3 times/month	50	2.0	1	1
Anal sex	Yes	74	10.8	1.4 (0.61–3.12)	1.0 (0.42–2.43)
	No	422	8.1	1	1
Oral sex	Yes	190	9.5	1.2 (0.65–2.33)	0.8 (0.39–1.58)
	No	306	7.8	1	1
Hormonal contraceptives	Yes	64	4.7	0.5 (0.15–1.65)	0.5 (0.15–1.90)
	No	432	9.0	1	1
Frequency condom use	Never	215	9.8	1.3 (0.71–2.52)	1.36 (0.70–2.64)
	Yes, at least sometimes	281	7.5	1	1

**% HSV-2:** Seroprevalence of Herpes Simplex Virus type; **ORc:** (Crude Odds Ratio), **ORa**: (Adjusted odds ratio, adjusted by Age, Trimester, Alcohol consumption, Antibiotic use, Frequency of sex and Frequency condom use).

## Data Availability

The datasets used and/or analyzed during the current study are available from the corresponding author on reasonable request.

## References

[1] WHO (2022). Herpes Simplex Virus. https://www.who.int/news-room/fact-sheets/detail/herpes-simplex-virus.

[2] Tata S., Johnston C., Huang M.L., Selke S., Magaret A., Corey L., Wald A. (2010). Overlapping reactivations of herpes simplex virus type 2 in the genital and perianal mucosa. J. Infect. Dis..

[3] Wald A., Corey L. (2007). Persistence in the population: Epidemiology, transmission. Human Herpesviruses: Biology, Therapy, and Immunoprophylaxis.

[4] Domercant J.W., Jean Louis F., Hulland E., Griswold M., Andre-Alboth J., Ye T., Marston B.J. (2017). Seroprevalence of Herpes Simplex Virus type-2 (HSV-2) among pregnant women who participated in a national HIV surveillance activity in Haiti. BMC Infect. Dis..

[5] Xu F., Markowitz L.E., Gottlieb S.L., Berman S.M. (2007). Seroprevalence of herpes simplex virus types 1 and 2 in pregnant women in the United States. Am. J. Obstet. Gynecol..

[6] Shannon C., Klausner J. (2018). The Growing Epidemic of Sexually Transmitted Infections in Adolescents: A Neglected Population. Curr. Opin. Pediatr..

[7] Gutierrez J.P., Bertozzi S.M., Conde-Glez C.J., Sanchez-Aleman M.A. (2006). Risk behaviors of 15–21 years old in Mexico lead to a high prevalence of sexually transmitted infections: Results of a survey in disadvantaged urban areas. BMC Public Health.

[8] Sánchez-Alemán M.A., Conde-Glez C.J., Gayet C., García-Cisneros S., Uribe-Salas F. (2005). Sexual behavior and herpes simplex virus 2 infection in college students. Arch. Med. Res..

[9] Shafii T., Burstein G.R. (2009). The adolescent sexual health visit. Obstet. Gynecol. Clin. N. Am..

[10] Schillinger J.A., McKinney C.M., Garg R., Gwynn R.C., White K., Lee F., Blank S., Thorpe L., Frieden T. (2008). Seroprevalence of herpes simplex virus type 2 and characteristics associated with undiagnosed infection: New York City, 2004. Sex. Transm. Dis..

[11] Garland S.M., Steben M. (2014). Genital herpes. Best Pract. Res. Clin. Obstet. Gynaecol..

[12] Pinninti S.G., Kimberlin D.W. (2013). Maternal and neonatal herpes simplex virus infections. Am. J. Perinatol..

[13] Straface G., Selmin A., Zanardo V., De Santis M., Ercoli A., Scambia G. (2012). Herpes simplex virus infection in pregnancy. Infect. Dis. Obstet. Gynecol..

[14] Bahena-Román M., Sánchez-Alemán M.A., Contreras-Ochoa C.O., Lagunas-Martínez A., Olamendi-Portugal M., López-Estrada G., Delgado-Romero K., Guzmán-Olea E., Madrid-Marina V., Torres-Poveda K. (2020). Prevalence of active infection by herpes simplex virus type 2 in patients with high-risk human papillomavirus infection: A cross-sectional study. J. Med. Virol..

[15] Herrera-Ortiz A., Conde-Glez C.J., Vergara-Ortega D.N., García-Cisneros S., Olamendi-Portugal M.L., Sánchez-Alemán M.A. (2013). Avidity of Antibodies against HSV-2 and Risk to Neonatal Transmission among Mexican Pregnant Women. Infect. Dis. Obstet. Gynecol..

[16] Sánchez-Alemán M.A., Uribe-Salas F.J., Lazcano-Ponce E.C., García-Cisneros S., Eguiza-Fano S., Conde-Glez C.J. (2010). HSV-2 seroincidence among Mexican college students: The delay of sexual debut is not enough to avoid risky sexual behaviours and virus transmission. Sex. Transm. Infect..

[17] Vergara-Ortega D.N., Sevilla-Reyes E.E., Herrera-Ortiz A., Torres-Ibarra L., Salmerón J., Lazcano-Ponce E., Sánchez-Alemán M.A. (2018). Real time PCR to evaluate HSV-2 shedding from anal and genital samples among men who have sex with men, living with HIV. J. Med. Virol..

[18] James C., Harfouche M., Welton N.J., Turner K.M., Abu-Raddad L.J., Gottlieb S.L., Looker K.J. (2020). Herpes simplex virus: Global infection prevalence and incidence estimates, 2016. Bull. World Health Organ..

[19] Sanchez-Aleman M.A., Del Villar-Tapia Y.G., Gutierrez J.P., Garcia-Cisneros S., Olamendi-Portugal M.L., Herrera-Ortiz A., Velazquez-Meza M., Conde-Glez C.J. (2018). Heterogeneity of Herpes Simplex Virus Type 2 Seroprevalence From a National Probability Survey in Mexico, 2012. Sex. Transm. Dis..

[20] DGE (2023). Anuario de Morbilidad 1984–2021. https://epidemiologia.salud.gob.mx/anuario/html/incidencia_enfermedad.html.

[21] Smith J.S., Robinson N.J. (2002). Age-specific prevalence of infection with herpes simplex virus types 2 and 1: A global review. J. Infect. Dis..

[22] Shin H., Iwasaki A. (2013). Generating protective immunity against genital herpes. Trends Immunol..

[23] Rosenberg M., Pettifor A., Van Rie A., Thirumurthy H., Emch M., Miller W.C., Gómez-Olivé F.X., Twine R., Hughes J.P., Laeyendecker O. (2015). The Relationship between Alcohol Outlets, HIV Risk Behavior, and HSV-2 Infection among South African Young Women: A Cross-Sectional Study. PLoS ONE.

[24] Gutierrez J.P., Conde-González C.J., Walker D.M., Bertozzi S.M. (2007). Herpes Simplex Virus Type 2 among Mexican High School Adolescents: Prevalence and Association with Community Characteristics. Arch. Med. Res..

[25] Kalinderi K., Delkos D., Kalinderis M., Athanasiadis A., Kalogiannidis I. (2018). Urinary tract infection during pregnancy: Current concepts on a common multifaceted problem. J. Obstet. Gynaecol..

[26] Birdthistle I., Floyd S., Nyagadza A., Mudziwapasi N., Gregson S., Glynn J.R. (2009). Is education the link between orphanhood and HIV/HSV-2 risk among female adolescents in urban Zimbabwe?. Soc. Sci. Med..

[27] Stoner M.C.D., Edwards J.K., Miller W.C., Aiello A.E., Halpern C.T., Julien A., Rucinski K.B., Selin A., Twine R., Hughes J.P. (2018). Does Partner Selection Mediate the Relationship Between School Attendance and HIV/Herpes Simplex Virus-2 Among Adolescent Girls and Young Women in South Africa: An Analysis of HIV Prevention Trials Network 068 Data. J. Acquir. Immune Defic. Syndr..

[28] Stoner M.C.D., Edwards J.K., Miller W.C., Aiello A.E., Halpern C.T., Julien A., Selin A., Hughes J.P., Wang J., Gomez-Olive F.X. (2017). Effect of Schooling on Age-Disparate Relationships and Number of Sexual Partners among Young Women in Rural South Africa Enrolled in HPTN 068. J. Acquir. Immune Defic. Syndr..

[29] Brown Z.A., Wald A., Morrow R.A., Selke S., Zeh J., Corey L. (2003). Effect of serologic status and cesarean delivery on transmission rates of herpes simplex virus from mother to infant. JAMA.

[30] Picone O. (2017). Genital herpes and pregnancy: Epidemiology, clinical manifestations, prevention and screening. Guidelines for clinical practice from the French College of Gynecologists and Obstetrician (CNGOF). Gynecol. Obstet. Fertil. Senol. Diciembre.

[31] Patel R., Kennedy O.J., Clarke E., Geretti A., Nilsen A., Lautenschlager S., Green J., Donders G., van der Meijden W., Gomberg M. (2017). 2017 European guidelines for the management of genital herpes. Int. J. STD AIDS.

[32] Gardella C., Brown Z.A., Wald A., Morrow R.A., Selke S., Krantz E., Corey L. (2005). Poor correlation between genital lesions and detection of herpes simplex virus in women in labor. Obstet. Gynecol..

[33] Gardella C., Huang M.L., Wald A., Magaret A., Selke S., Morrow R., Corey L. (2010). Rapid polymerase chain reaction assay to detect herpes simplex virus in the genital tract of women in labor. Obstet. Gynecol..

[34] (2020). Management of Genital Herpes in Pregnancy: ACOG Practice Bulletin, Number 220. Obstet. Gynecol..

[35] Samies N.L., James S.H. (2020). Prevention and treatment of neonatal herpes simplex virus infection. Antivir. Res..

